# GraphSAW: A web-based system for graphical analysis of drug interactions and side effects using pharmaceutical and molecular data

**DOI:** 10.1186/s12911-015-0139-5

**Published:** 2015-02-28

**Authors:** Alban Shoshi, Tobias Hoppe, Benjamin Kormeier, Venus Ogultarhan, Ralf Hofestädt

**Affiliations:** Bioinformatics/Medical Informatics Department, Bielefeld University, Universitätsstraße 25, 33615 Bielefeld, Germany

**Keywords:** Medical informatics, Information system, Decision support system, Drug therapy, Adverse drug interaction, Adverse drug reaction, Drug side effects

## Abstract

**Background:**

Adverse drug reactions are one of the most common causes of death in industrialized Western countries. Nowadays, empirical data from clinical studies for the approval and monitoring of drugs and molecular databases is available.

**Methods:**

The integration of database information is a promising method for providing well-based knowledge to avoid adverse drug reactions. This paper presents our web-based decision support system GraphSAW which analyzes and evaluates drug interactions and side effects based on data from two commercial and two freely available molecular databases. The system is able to analyze single and combined drug-drug interactions, drug-molecule interactions as well as single and cumulative side effects. In addition, it allows exploring associative networks of drugs, molecules, metabolic pathways, and diseases in an intuitive way. The molecular medication analysis includes the capabilities of the upper features.

**Results:**

A statistical evaluation of the integrated data and top 20 drugs concerning drug interactions and side effects is performed. The results of the data analysis give an overview of all theoretically possible drug interactions and side effects. The evaluation shows a mismatch between pharmaceutical and molecular databases. The concordance of drug interactions was about 12% and 9% of drug side effects. An application case with prescribed data of 11 patients is presented in order to demonstrate the functionality of the system under real conditions. For each patient at least two interactions occured in every medication and about 8% of total diseases were possibly induced by drug therapy.

**Conclusions:**

GraphSAW (http://tunicata.techfak.uni-bielefeld.de/graphsaw/) is meant to be a web-based system for health professionals and researchers. GraphSAW provides comprehensive drug-related knowledge and an improved medication analysis which may support efforts to reduce the risk of medication errors and numerous drastic side effects.

## Background

In recent decades the development of highly effective drugs has crucially marked scientifically-based medicine. According to recent studies every fifth medication is incorrect and every fourteenth medication is potentially dangerous [1]. Over ten years it has been known that 1% of medication errors lead to adverse drug reactions (ADRs) [2,3] and are a common cause of death [4]. In 2001, a study by Ebbesen et al. [5] provided reliable data on such deaths. They investigated 732 deaths among 13,992 patients over a treatment period of two years with regard to the cause of death and showed that 0.95% of patients in a department of internal medicine suffered fatal adverse drug events (ADEs). Autopsy results and postmortem blood level measurements used for identifying the exact cause of death showed for the first time that almost half of these deaths were due to avoidable drug-drug interactions and dosage errors [5]. In case of Germany, a conservative extrapolation leads to tens of thousands of deaths per year as a consequence of medication errors [6].

Computerized physician order entry systems (CPOES) and computerized decision support systems (CDSS) assist in the prescription of drugs and indicate potentially serious drug interactions as well as overdoses [7,8]. The risk check of available patient data and their respective drugs is based on pharmaceutical databases. Several interaction databases, including ABDA [9], MediQ [10], DrugDex [11] and AiDKlinik® [12], were evaluated by the hospital Klinikum Dachau with regard to the retrieval rate and relevance of drug interactions. A practical test [13] showed that all theoretically possible drug interactions described in literature cannot be found within only one database. For a reliable retrieval it is necessary to check drug interactions by using at least two databases.

In the recent past a variety of commercial and non-commercial drug-related databases have been established. Despite the large amount of drug-related information, the optimization of multi-medications using additional molecular data remains still complicated. These databases can however help to identify uncovered patient risk factors in the patient-specific drug therapy and to exclude certain drugs for patient treatment (e.g., if there is a serious drug-drug interaction or if the patient's metabolic profile does not respond to a specific drug).

Appropriate systems for health professionals and researchers are required to achieve substantial improvements in drug prescription quality for above reasons. For this purpose we have developed a web-based system named GraphSAW which integrates drug-related pharmaceutical and molecular databases. This combination enables GraphSAW to provide a discovery toolkit for analysis and visualization of drug interactions and side effects.

### Related work

To meet the challenges of drug therapy safety, we evaluated three related web-based systems to expose their advantages and disadvantages.

### Promiscuous

Promiscuous [14] was developed to facilitate the progress of drug repositioning. The system includes three different entities: drugs, proteins, and side effects. Information about the relations of these entities was gathered from different molecular databases. Additional information was extracted and manually curated from PubMed publications. All data was integrated into the independent Promiscuous database. The system provides search forms for drug-protein interactions, drug side effects, and drug-pathway interactions. The results are shown in an interactive network and listed in a tabular form. Note that, there is no possibility for determining drug-drug interactions.

### Stitch

The search tool for interactions of chemicals, short STITCH [15], is a database of interaction data and an interactive web tool for the exploration of interaction networks of proteins and chemicals, including drugs. The basis of this system consists of chemical compounds from PubChem which were mapped on thirteen molecular databases. In addition, a confidence-value is assigned to each interaction relation based on the reliability of the data source. The results are visualized in an interactive network with three different views: confident-view, evidence-view, and action-view. Further information of the network is shown in a table. Information about metabolic pathways and drug side effects are not included in STITCH.

### Kegg medicus

KEGG MEDICUS [16] is an integrated information resource of diseases, drugs, and health-related substances, aiming to bring the genomic revolution of society. KEGG MEDICUS currently integrates the KEGG DRUG and KEGG DISEASE databases, human pathways and drug development pathways in KEGG PATHWAY database, and FDA drug labels for both prescription and OTC drugs in the USA and Japan. Given a list of drugs, the Drug Interaction Checker reports any drug-drug interactions associated with contraindications and precautions. The information is computerized in lists. Network-based information and visualization about drug side effects and drug-induced diseases is missing.

As a concluding remark, the evaluation showed that all presented systems were inappropriate for analyzing multi-medications and avoiding adverse drug reactions. They provided only functionalities either for drug-drug interactions or drug side effects. Furthermore, the systems were using only molecular drug-related databases. A commercial, pharmaceutical database such as ABDA, which contains approved and validated drug-related data, can ensure better data quality and more comprehensive knowledge. Moreover, an interaction network can simplify the understanding of drug effects in the human organism. Because of these facts, a new system was required. This new system should offer functionalities for analyzing drug side effects and drug interactions at different pharmaceutical and molecular levels. It should offer insight into underlying biochemical networks where drugs and molecules interact in the same metabolic pathway. Therefore, the system should offer an associative network and a tabular view in order to visualize the coherency of results in an intuitive way.

## Methods

**Figure 1:**
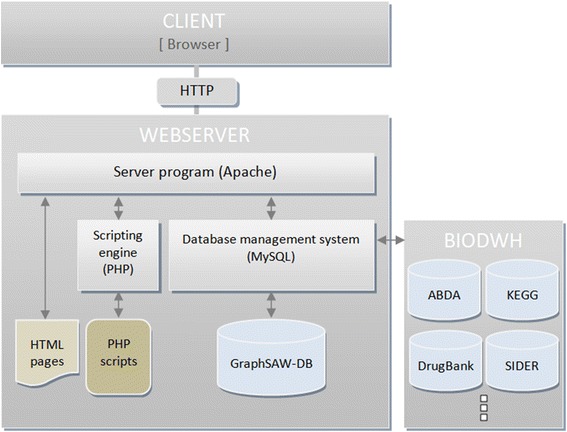
**Client/Server architecture of the system GraphSAW.** The client communicates via Internet with a central server and the biological data warehouse BioDWH. On the server side, an Apache HTTP Web server and PHP scripts to dynamically generated static HTML pages are used. The integrated databases are listed in the BioDWH.

GraphSAW was developed as a user-friendly decision support system for health professionals and researchers and is based on common client/server architecture (Figure 1). It is a web-based system that is platform independent and accessible via the Internet. The application logic was implemented in PHP, ^a^ HTML, ^b^ and CSS ^c^. The core of GraphSAW represents the database, which integrates drug-related pharmaceutical and molecular databases. Due to this comprehensive knowledge GraphSAW provides a discovery toolkit for analysis and visualization. JavaScript components for associative network visualization were developed for a scientific yet intuitive way of analyzing and exploring the data. Therefore, jQuery and Information Visualization Toolkit library (InfoVis) [17] were used.

An arbitrary set of user inputs are sent via an asynchronous HTTP-request to the web server. On the server side, the results of the database evaluation are converted with PHP scripts into JSON format and transferred to the InfoVis library which renders the graph for visualizing the results.

### Data integration

GraphSAW contains six different types of entities: drugs, interactions, side effects, molecules, diseases, and pathways. The entities are connected to each other through drug-drug interaction, drug-side effects, drug-molecule, drug-disease, drug-pathway, and pathway-disease relations. To provide a comprehensive dataset, the information was retrieved from two commercial and two freely available databases: ABDA [9], KEGG [18], SIDER [19], and DrugBank [20], whereas the latter represents the basis of the system. DrugBank is the largest resource that collects binding data on small molecules, in particular those of drugs and proteins. A total number of 6711 approved and experimental drugs were extracted and mapped to additional databases. Moreover, DrugBank contains drug-drug interactions as well as drug-protein interactions combined with the DrugBank Partners database. Further drug interactions were obtained from the commercial database ABDA that is based on approved and validated drug-related data in comparison to DrugBank. ABDA contains comprehensive facts for dealing with more than 47,000 drugs such as information about application and composition, risks and drug interactions. Codes of ATC [21], an **A**natomical **T**herapeutical **C**hemical classification system, were used to classify drugs of the databases DrugBank and ABDA. These DrugBank drugs were assigned to the ABDA drugs by ATC-codes and identifiers. The ABDA database includes also the side effects of drugs. More than 4500 side effects (3,135 different; 1,381 synonyms) were extracted automatically from full-text information in German and translated manually into English. An additional 4,192 different drug side effects were obtained from SIDER. Terms of MedDRA ^d^ [22], a unified and standardized medical terminology, were used for coding drug side effects of both databases. The mapping between drugs of DrugBank and SIDER was realized by drug names because these databases did not have corresponding identifiers for substances. By drug mapping, the interactions and side effects were assigned to drugs of all databases. Information about metabolic pathways was obtained from KEGG, which already integrates substances from DrugBank [20], PubChem [23], CAS [24], LigandBox [25], and NIKKAJI [26]. DrugBank identifiers were used for the mapping and merging of these data sources.

All databases were integrated by implementing SAX ^e^ -Parser in Java and the bio data warehouse BioDWH [27]. BioDWH is a novel bioinformatics data warehouse software kit that integrates biological information from multiple public life science data sources into a local database management system. It differs from other approaches by providing up-to-date integrated knowledge, platform and database independence as well as high usability and customization. This way of integration provides the transformation from different data file types such as XML or TSV into MySQL databases. Using the data warehouse architecture also ensures both the availability as well as the relevance of the data sources.

An additional GraphSAW database was created to store meta data such as extracted and translated side effects from ABDA. It also contains lists of drugs for a “so-called” auto-complete functionality. This feature is used to suggest possible drugs to users, which guarantee search results.

### Data analysis and visualization

**Figure 2:**
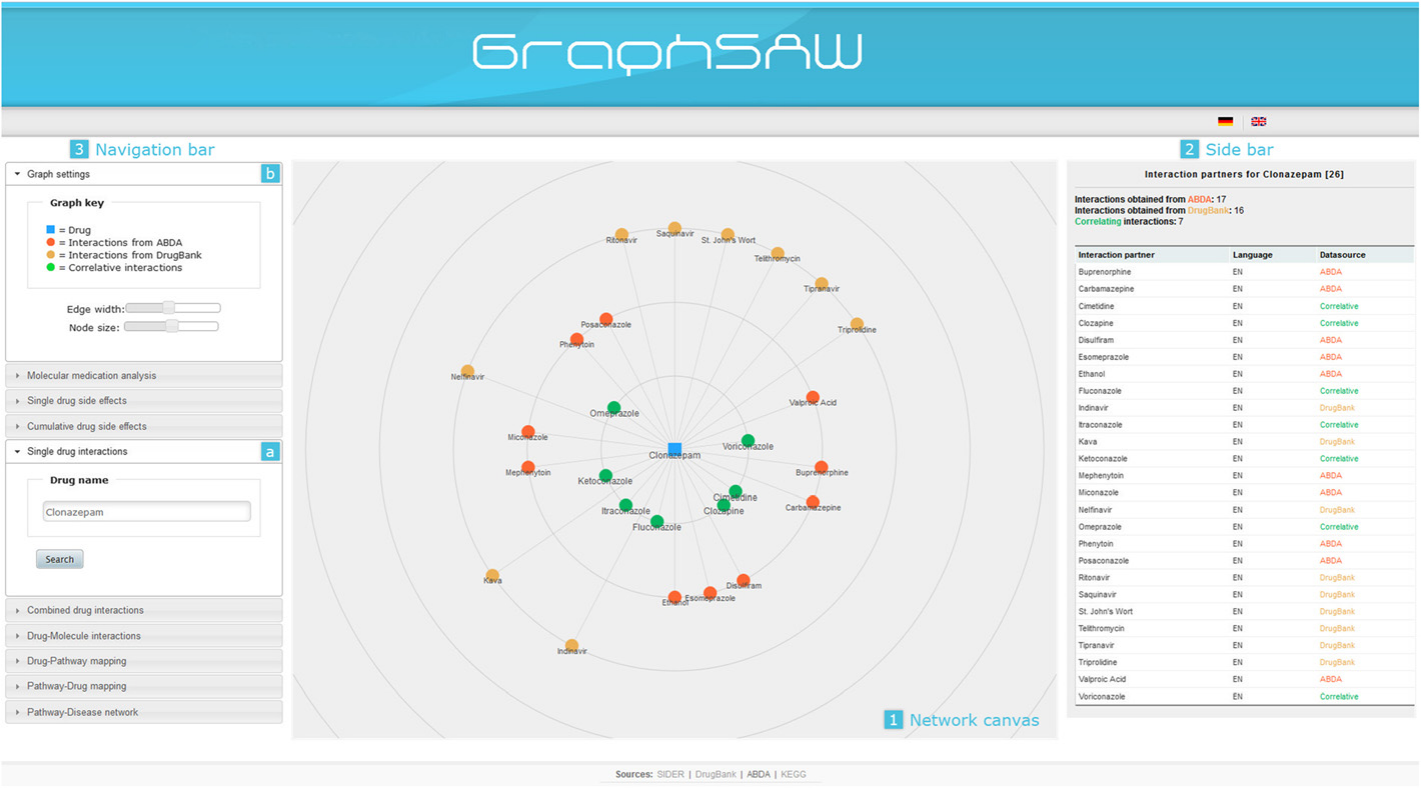
**Network visualization interface of GraphSAW explaining „Single drug interactions“ for “Clonazepam”.** The library generates a radial network, thus, a central drug node encircled by color-coded interacting drugs at higher levels (1). On the first circle, correlating interactions from ABDA and SIDER are placed and colored in green. On the second circle, drug-drug interactions from ABDA (red), and on the last circle, drug-drug interactions from SIDER (yellow) are shown. Detailed information about nodes and edges are available via the bar on the right side (2). Furthermore, user inputs in the search form (a) and basic graph settings (b) can be adjusted in the navigation bar (3) on the left side.

The user interface represents database entities (drugs, side effects, molecules, diseases, and pathways) as nodes in a network with edges representing the relations between them. Starting from an arbitrary set of user inputs, the system analyzes the integrated knowledge and makes an evaluation in terms of risks (Figure 2). The library generates a radial network, thus, one or a couple of central nodes represent the inputs encircled by color-coded results at higher levels (1). The radial network is used to separate the results at different levels depending on different databases. Detailed information about nodes and edges are available as a color-coded plain list via the bar on the right side (2). Furthermore, user inputs in the search form (a) and basic graph settings (b) can be adjusted in the navigation bar (3) on the left side.

The data delivered by GraphSAW can be queried through various search forms which are described in more detail in the following. GraphSAW provides three types of drug analysis: drug interactions and drug side effects, which are based on pharmaceutical and molecular data, and the molecular medication analysis which is based on molecular data.

#### Analysis of drug interactions

Multi-medications are associated with a significantly increased risk for adverse drug reactions and presents health professionals with a major challenge of adequate prescription. Drug interactions can be determined at three different levels: drug-drug, drug-molecule, and drug-pathway interactions.

At the first level, search forms for *single and combined drug interactions* were implemented in GraphSAW. *‘Single drug interactions’* enables users to search for interaction partners of a single drug. Interaction partners are merged, compared, ordered alphabetical and visualized depending on the databases ABDA and DrugBank. *’Combined drug interactions’* is one of the main features of the system. This feature allows users to check their multi-medications for interactions. Both pharmaceutical and molecular drug-drug interactions are retrieved from ABDA and DrugBank. Interacting drugs are placed as color-coded nodes in the network with edges representing the interactions between them.

*Drug-molecule interactions* can be queried at the second level. Especially the family of Cytochrome P450 Enzymes (CYPs) plays an important role in the degradation of most drugs. Many drugs inhibit or induce the activity of Cytochrome P450 Enzymes (CYPs) which is important to health professionals trying to give an appropriate dosage of those drugs. If a drug induces a CYP that is also active in another drug’s metabolism, the dosage of the first drug must be enhanced to achieve a therapeutic effect. In the case of the inhibition of a CYP, the dosage of the drug can be reduced which also decreases side effects. *Interactions between drugs and molecules* (targets, enzymes, transporter, and carrier proteins) can be queried by drug name. This information is retrieved from DrugBank and DrugBank Partners.

At the last level, *drug-pathway* and *pathway-disease networks* can also be explored by GraphSAW. This drug mapping on metabolic pathways or vice versa was implemented in order to put the entities in a biochemical context. This information is retrieved from KEGG (DrugBank, PubChem, CAS, LigandBox, and NIKKAJI). Drugs of a chosen database are mapped and visualized on an entered metabolic pathway. Moreover, the total number of result hits and matching results of the remaining databases are shown in the side bar. The effects of drugs can only be fully understood by considering a *pathway-disease network*, which includes drugs, metabolic pathways, and diseases. The metabolic network maps are probably the most comprehensive of all biological networks. The easiest way to search in such a complex network is by using the name of a pathway.

#### Analysis of drug side effects

Knowledge about drug side effects can be extremely useful in supporting the quality of prescribing medications. Similar to drug interactions, search forms for *single and cumulative drug side effects* were implemented. *‘Single drug side effects’* enables users to search for side effects of a single drug. Package inserts of prescription drugs (ABDA) are supposed to contain side effects but such information is not necessarily complete. Therefore, the side effects are merged, compared, ordered alphabetical and visualized depending on the databases ABDA and SIDER. *Cumulative drug side effects* are side effects that occur in at least two of the prescribed drugs. This means that the occurrence probability of cumulative side effects increases with the number of prescribed drugs. Therefore, they represent a particularly high risk – especially for long-term treatments with psychotropic drugs. With this feature, cumulative side effects of entered drugs can be identified and avoided.

#### Molecular medication analysis

**Figure 3:**
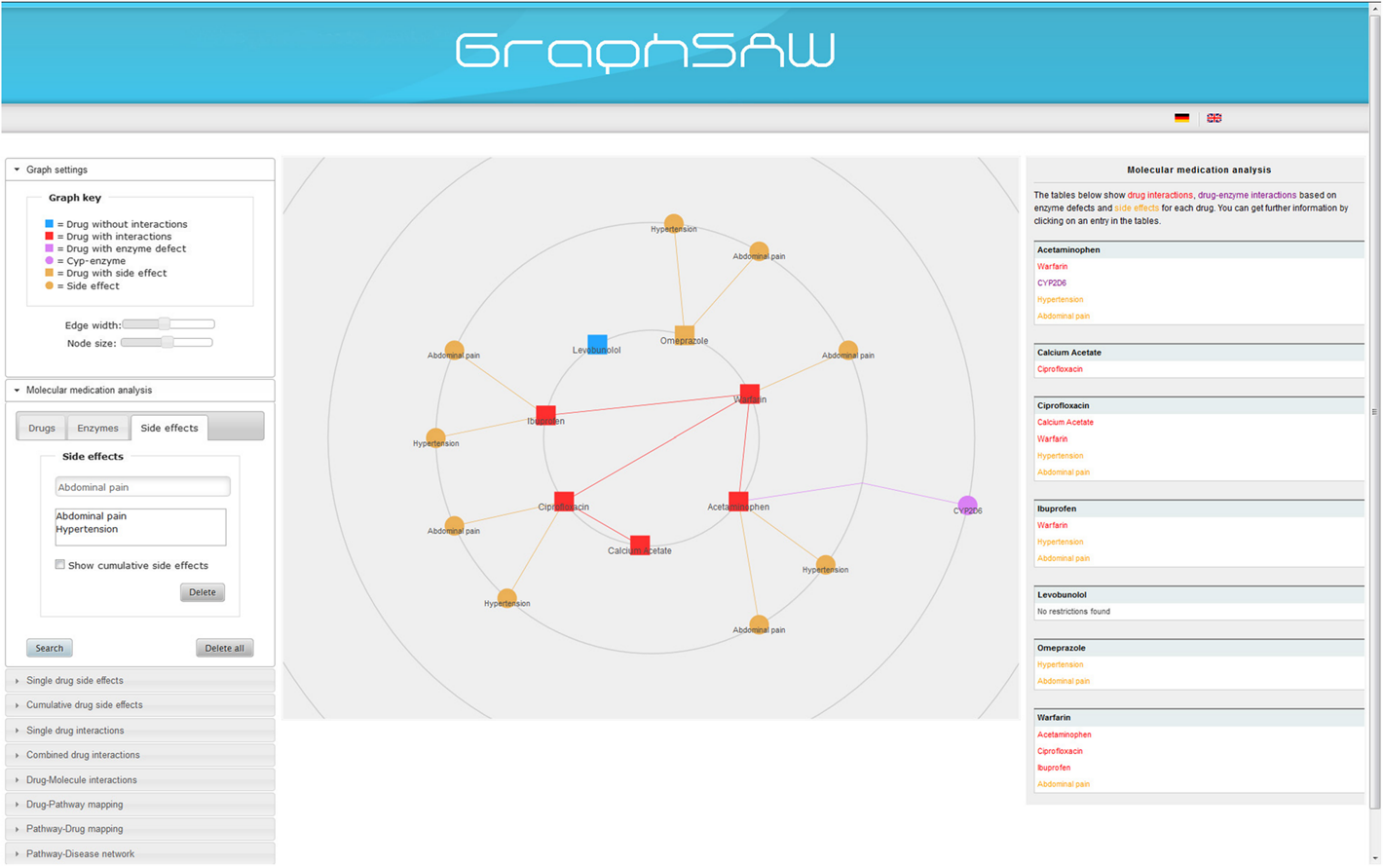
**Network visualization interface of GraphSAW explaining “molecular medication analysis” for seven drugs and two side effects.** The main window enables users to view the network of drug-drug interactions (red), drug-enzyme interactions (violet), drug side effects (yellow), and drugs without potential risks (blue). The right side bar shows context sensitive information about nodes and edges.

The *‘molecular medication analysis’* includes the previously mentioned search forms *‘combined drug interactions’*, *‘drug-molecule interactions’*, and *‘drug side effects’*. This main feature allows users to check the medications for molecular drug side effects, drug-drug and drug-enzyme interactions. Therefore, users only need to enter the prescribed drugs, occurred side effects or drug-induced diseases, and the defect Cytochrome P450 enzymes (CYP) into the system. As an example, Figure 3 shows the result of the molecular medication analysis for seven drugs and two side effects. Starting from this arbitrary set of user inputs, the system checks whether a drug interacts with another drug or causes side effects. Further information like the effect of the interaction between two drugs, the frequency of a side effect, and the activity of the defect CYP can be queried.

### Statistical evaluation and application case

The variety of possible drug interactions and side effects are not tallied systematically and are hardly manageable, even for experts. GraphSAW gives insight into the distribution of the integrated pharmaceutical and molecular data concerning drug interactions and drug side effects. Furthermore, an analysis of leading drugs (top 20) was carried out, i.e., according to the number of prescriptions made in 2008, in terms of drug interactions and side effects. This data was obtained from the “GEK-Arzneimittel-Report 2009” [28]. This report of the German health insurance GEK evaluates the results of drug data in the period from 2007 to 2008. The drug prescriptions include output drugs in pharmacies paid by the GEK. Finally, an application case with real life patient data was performed in order to analyze multi-medications for drug interactions and drug-induced diseases. The patient data was provided by a medical practice.

### Statistical evaluation of data distribution

**Figure 4:**
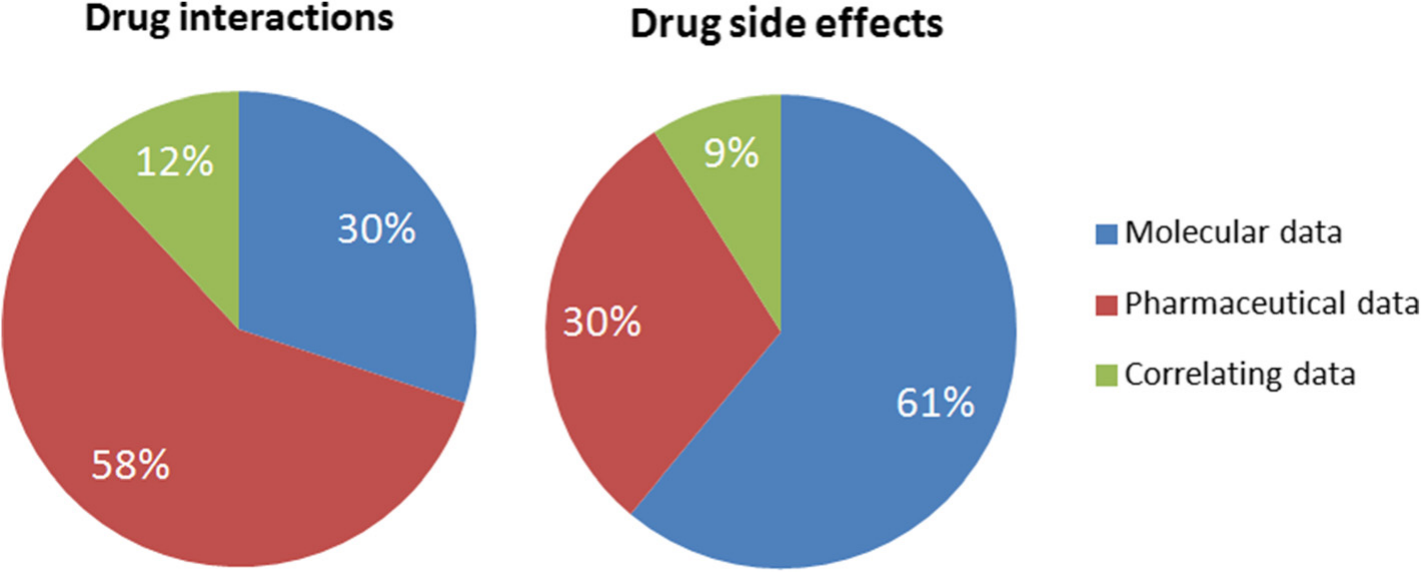
**Data distribution of drug interactions and side effects.** Number of drug interactions and side effects retrieved from pharmaceutical and molecular data.

The distribution of drug interactions and drug side effects data within the integrated pharmaceutical and molecular databases was analyzed. As summarized in Figure 4, the analysis has revealed a total of 51,520 drug interaction hits, whereas 29,854 (58%) of the total interactions are recorded in ABDA and 15,518 (30%) in DrugBank. Furthermore, only 6,148 (12%) of total interactions are shared by both databases. As a consequence, about 30% of total drug interactions are missing in computerized decision support systems which are based on pharmaceutical data of the ABDA database.

The analysis of drug side effects has revealed an even more surprising result. The number of total side effect hits is 140,651 whereas 42,213 (30%) of the total side effects are recorded in ABDA and 85,873 (61%) in SIDER. The number of correlative side effect hits is 12,565 (9%). As a consequence, more than 60% of total side effects are missing in ABDA. Similar to drug-drug interactions only a small part of side effects are shared by both databases.

### Statistical evaluation of drug interactions

**Figure 5:**
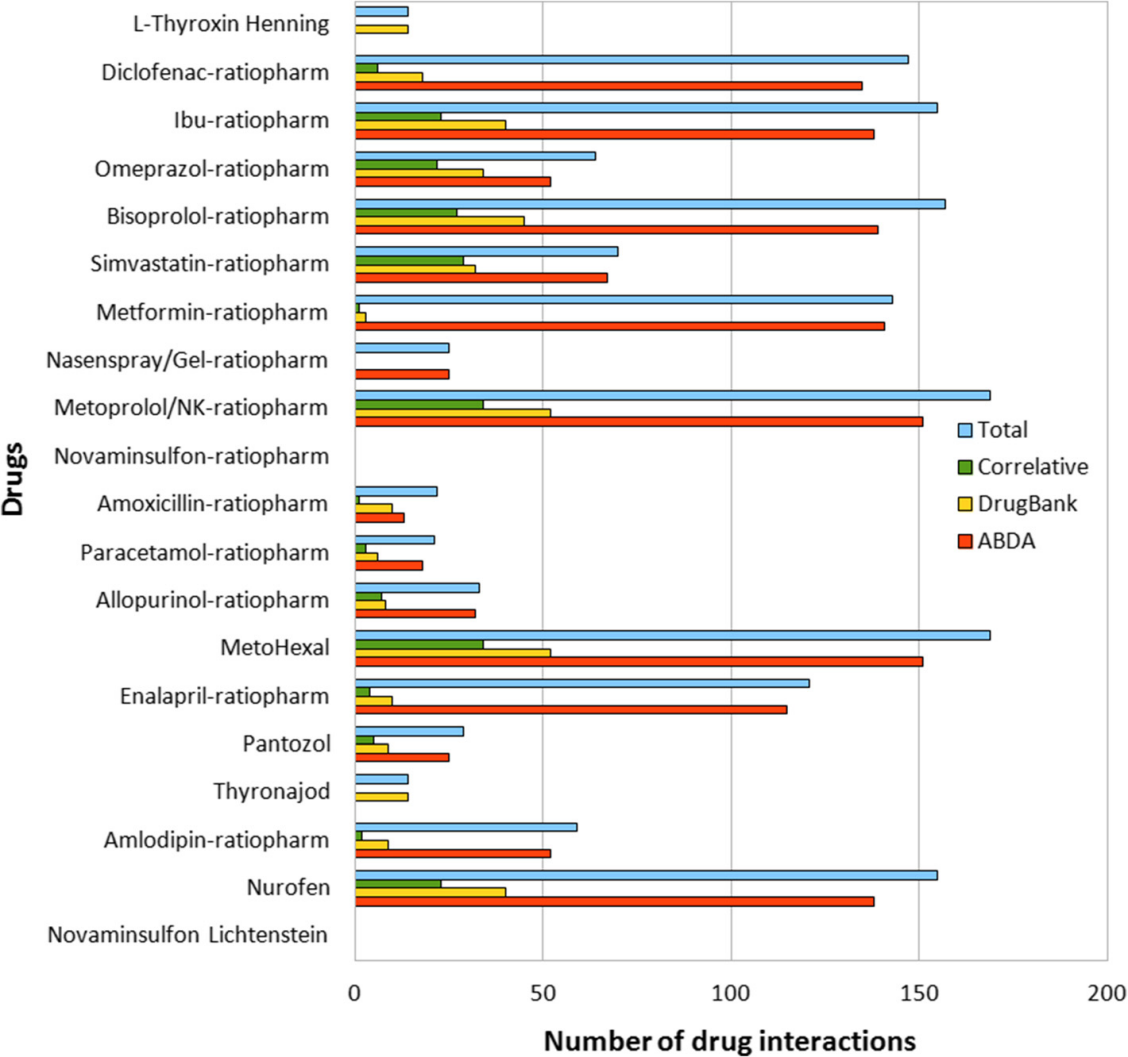
**Number of drug interactions.** Number of drug interactions retrieved from the databases ABDA and DrugBank in correlation to the top 20 drugs.

Figure 5 describes the number of identified drug interactions of the top 20 drugs. The current analysis has revealed a total of 1,567 interaction hits whereas 1,392 (88.83%) of the total interactions are recorded in ABDA and 396 (25.27%) in DrugBank. The number of correlative interaction hits is 221 (14.10%), representing the overlap of both databases, while 1,346 hits (85.90%) are non-correlative. Consequently, 75.28% of the overall 1,788 interaction hits recorded from both databases are not matched with each other.

### Statistical evaluation of drug side effects

**Figure 6:**
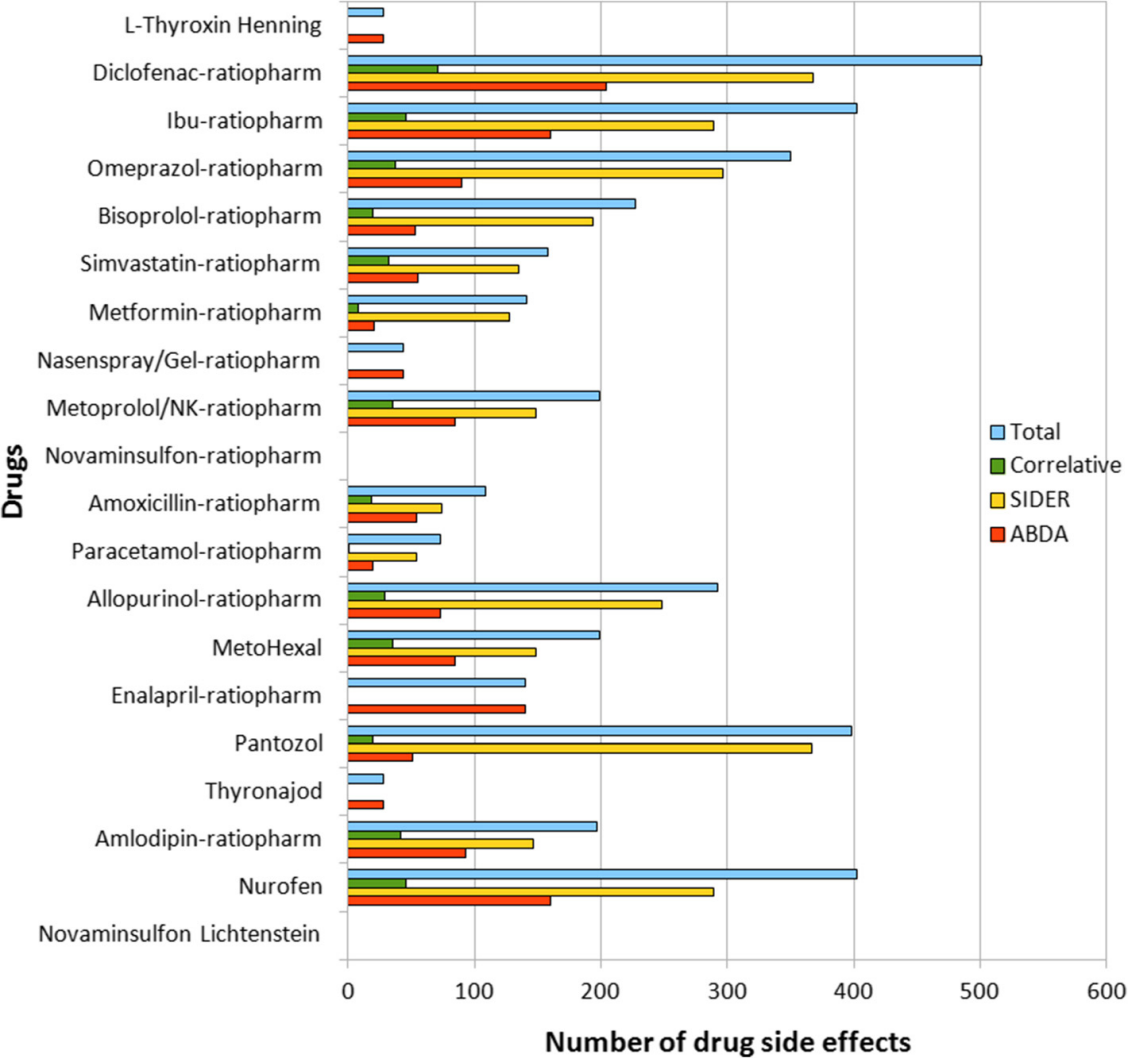
**Number of drug side effects.** Number of drug side effects retrieved from the databases ABDA and SIDER in correlation to the top 20 drugs.

Figure 6 shows the number of identified side effects of the top 20 drugs. The number of total side effect hits is 3,890 whereas 1,444 (37.12%) of the total side effects are recorded in ABDA and 2,887 (74.22%) in DrugBank. The number of correlative interaction hits is 441 (11.34%) and 3,449 hits (88.66%) are non-correlative. As a consequence, it was revealed that 79.64% of the overall 4.331 side effect hits from both databases are not matched with each other.

### Application case

The following application case with real life data of eleven patients demonstrates the GraphSAW system under real conditions. The data was collected and provided by a medical practice. The patient’s data was collected from 10 men and 1 woman with an average age of 78 years and an average weight of 87 kg. These patients were diagnosed with 8 to 18 diseases and were treated with 5 to 17 multiple drugs.

**Figure 7:**
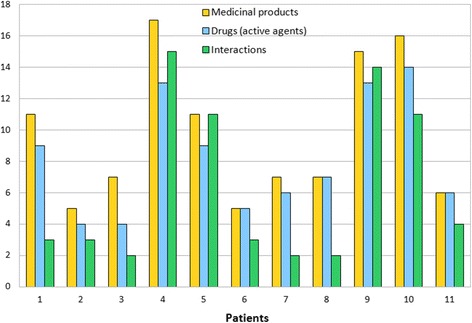
**Number of medicinal products, drugs, and interactions.** Number of medicinal products, drugs (active agents), and interactions for each patient.

The analysis of drug-drug interactions showed that at least two interactions occured in every medication of each patient. This bears a high potential for the occurrence of adverse drug reactions. Figure 7 shows the number of medicinal products, drugs (active agents), and drug interactions for each patient.

**Figure 8:**
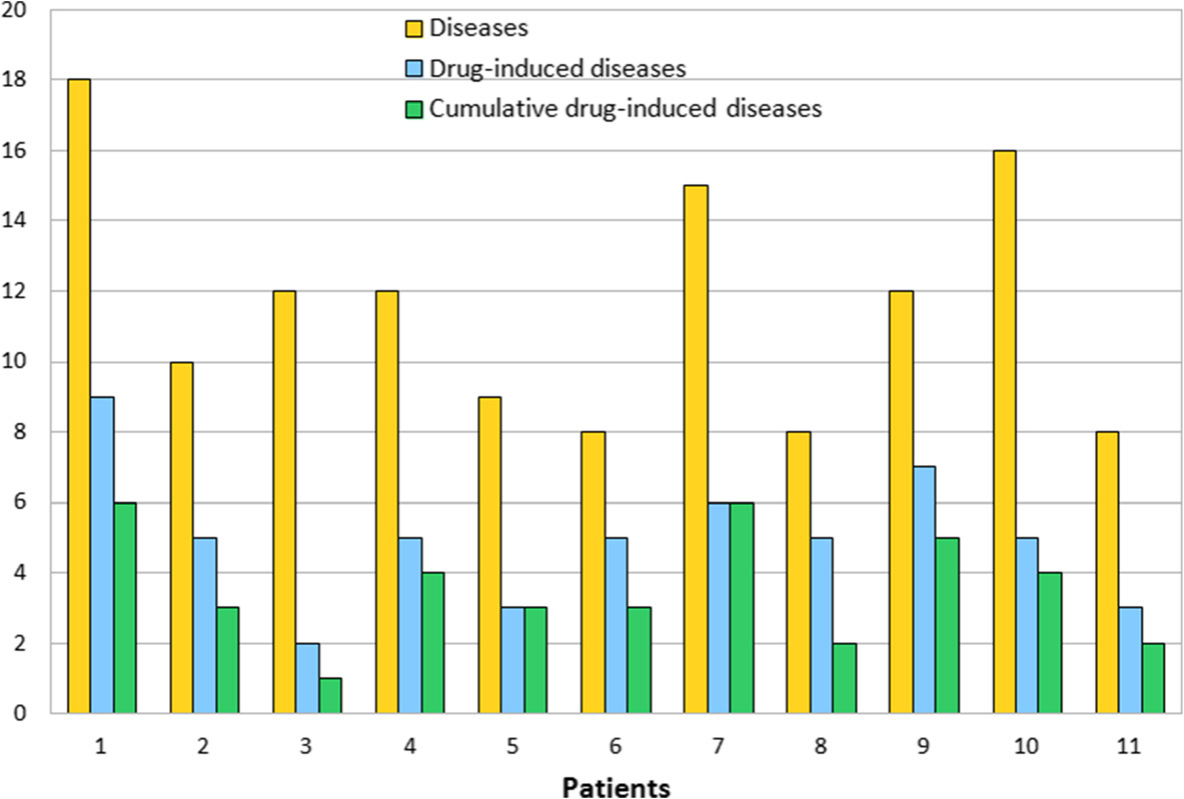
**Number of drug-induced diseases.** Number of all diseases, drug-induced and cumulative drug-induced diseases for each patient.

Even more important is the prevention of diseases which can be induced by drug therapy. The occurrence probability of these drug-induced diseases increases with the number of drugs sharing the same side effects. As summarized in Figure 8, 8.43% of total diseases were possibly induced by drug therapy. 70% of these drug-induced diseases occured only at more than one drug. This result leads one to assume that the number of diseases for each patient can be reduced by prescribing fewer drugs.

The datasets of the patients and all results of the analyses are available at http://tunicata.techfak.uni-bielefeld.de/graphsaw/application_case/index.html

## Results

The statistical evaluation of data distribution showed a mismatch between pharmaceutical and molecular databases. The concordance of drug interactions was about 12% and 9% of drug side effects. More than 30% of the molecular drug interactions and more than 61% of the molecular side effects were missing in the pharmaceutical database. Therefore, they are also missing in most computerized decision support systems. These results were confirmed by the analysis of the top 20 drugs on the German market. The concordance of drug interactions was about 14% and 11% of drug side effects. In addition, more than 35% of the molecular interactions and more than 60% of the molecular side effects were not recorded in the pharmaceutical database. Finally, the application case showed that for each patient at least two interactions occured in every medication. Moreover, about 8% of total diseases were possibly induced by drug therapy.

## Discussion

Although drugs are essential for the prevention and proper treatment of diseases, multi-medications still may cause serious adverse drug reactions which are usually known. Reliability on the extent and quality of information used by medical doctors for decision making lies at the heart of efforts towards improving prescription quality. GraphSAW provides users a graphical view on a well-based knowledge to analyze drug cocktails for adverse drug reactions and drug-induced diseases. The obtained results can be used to compare the risk of disease against the therapeutic risk (benefit/risk relation), i.e., to avoid the risk of under-treatment of patients which is possibly an even greater risk. GraphSAW will be extended by visualizing additional information such as interaction profiles (mechanism, effect, etc.) or side effect profiles (extent, frequencies, etc.). As adverse drug reactions do not occur in each patient in the same extent due to inter-individual genetic differences, a new feature to analyze polymorphisms is planned to be implemented.

## Conclusions

The drugs risks-check of decision support systems is usually based on pharmaceutical databases. Despite the large amount of drug-related information, the analysis and optimization of multi-medications using additional molecular data is still complicated and even for experts unmanageable. With GraphSAW we presented a new decision support system which integrates pharmaceutical and molecular databases. Based on this comprehensive knowledge, GraphSAW provides a toolkit for analysis and visualization. Users can check their medications for single and combined drug interactions, drug-molecule interactions as well as single and cumulative side effects. Moreover, the implemented visualization toolkit allows exploring associative networks of drugs, molecules, metabolic pathways, and diseases to fully understand effects of drugs in an intuitive way. Along this line the dosage of drugs can be adjusted to the protein balance of the patient, resulting in better drug tolerability. For further support, the molecular medication analysis includes the features of combined drug interactions, drug-molecule interactions, and drug side effects at the molecular level.

In conclusion, substantial improvements in patient safety can be achieved by using GraphSAW which analyzes multi-medications and evaluates with regards to pharmaceutical and molecular adverse drug reactions. Potential drug interactions, drug side effects, and drug-induced diseases can be avoided. Health professionals and researchers gain more comprehensive knowledge and can contribute to a patient-related analysis of medication with the features offered by GraphSAW.

### Endnotes

^a^PHP: Hypertext Preprocessor.

^b^Hypertext Markup Language.

^c^Cascading Style Sheets.

^d^Medical Dictionary for Regulatory Activities.

^e^SAX is the Simple API for XML. SAX was the first widely adopted API for XML in Java.

### Availability and requirements

GraphSAW is available at http://tunicata.techfak.uni-bielefeld.de/graphsaw/. Access to the system is restricted by the commercial databases and will be enabled by a request to the authors. To access all features of the system, the latest browser versions such as Internet Explorer 9+ or Mozilla Firefox 23+ should first be installed.
